# Impact of a Bayesian penalized likelihood reconstruction algorithm on image quality in novel digital PET/CT: clinical implications for the assessment of lung tumors

**DOI:** 10.1186/s40658-018-0223-x

**Published:** 2018-09-26

**Authors:** Michael Messerli, Paul Stolzmann, Michèle Egger-Sigg, Josephine Trinckauf, Stefano D’Aguanno, Irene A. Burger, Gustav K. von Schulthess, Philipp A. Kaufmann, Martin W. Huellner

**Affiliations:** 1 Department of Nuclear Medicine, University Hospital Zurich/University of Zurich, Ramistrasse 100, 8091 Zurich, Switzerland; 2 Department of Pathology and Molecular Pathology, University Hospital Zurich/University of Zurich, Zurich, Switzerland; 3 GE Medical Systems (Schweiz) AG, Glattbrugg, Switzerland

**Keywords:** Positron-emission tomography, Lung cancer, Image reconstruction, PET/CT, Image quality enhancement

## Abstract

**Background:**

The aim of this study was to evaluate and compare PET image reconstruction algorithms on novel digital silicon photomultiplier PET/CT in patients with newly diagnosed and histopathologically confirmed lung cancer. A total of 45 patients undergoing 18F-FDG PET/CT for initial lung cancer staging were included. PET images were reconstructed using ordered subset expectation maximization (OSEM) with time-of-flight and point spread function modelling as well as Bayesian penalized likelihood reconstruction algorithm (BSREM) with different *β*-values yielding a total of 7 datasets per patient. Subjective and objective image assessment with all image datasets was carried out, including subgroup analyses for patients with high dose (> 2.0 MBq/kg) and low dose (≤ 2.0 MBq/kg) of 18F-FDG injection regimen.

**Results:**

Subjective image quality ratings were significantly different among all different reconstruction algorithms as well as among BSREM using different *β*-values only (both *p* < 0.001). BSREM with a *β*-value of 600 was assigned the highest score for general image quality, image sharpness, and lesion conspicuity. BSREM reconstructions resulted in higher SUV_max_ of lung tumors compared to OSEM of up to + 28.0% (*p* < 0.001). BSREM reconstruction resulted in higher signal-/ and contrast-to-background ratios of lung tumor and higher signal-/ and contrast-to-noise ratio compared to OSEM up to a *β*-value of 800. Lower *β*-values (BSREM_450_) resulted in the best image quality for high dose 18F-FDG injections, whereas higher *β*-values (BSREM_600_) lead to the best image quality in low dose 18F-FDG PET/CT (*p* < 0.05).

**Conclusions:**

BSREM reconstruction algorithm used in digital detector PET leads to significant increases of lung tumor SUV_max_, signal-to-background ratio, and signal-to-noise ratio, which translates into a higher image quality, tumor conspicuity, and image sharpness.

## Background

Lung cancer is today the most common cause for cancer mortality, with an estimated 234,030 new cases occurring in the USA alone in 2018 [1]. Positron-emission tomography (PET) allows for imaging and quantitation of radiotracer uptake in vivo and may thereby visualize physiologic and pathophysiologic processes in patients [2]. For instance, 18F-fluorodeoxyglucose (18F-FDG) PET may be used to detect and quantify increased glucose metabolism in neoplastic lesions, such as primary tumors, lymph node metastases, or distant metastases [3]. Computed tomography (CT) enables a detailed assessment of local lung tumor extent, owing to its comparably higher spatial resolution [4, 5]. Therefore, hybrid imaging with 18F-FDG PET/CT has evolved as an important tool for comprehensive staging of lung cancer patients and is reimbursed by health insurances in many countries worldwide [6].

However, there are two main limitations of PET: first, the relatively low spatial resolution which may affect images both visually and quantitatively [7] and second, the generally relatively low signal-to-noise ratio [8]. There have been several technical advances within the last decade, including new hardware features, such as time-of-flight (TOF) acquisition [9] and silicon-based photodetectors (SIPM) as well as advanced image reconstruction methods, leading to an overall improvement of PET images. Iterative reconstruction methods have been widely adopted, replacing the initially used filtered back projection technique due to decreased artifacts and image noise [10]. Additionally, new reconstruction techniques, such as ordered subset expectation maximization (OSEM) and block sequential regularized expectation maximization (BSREM), came into clinical use and lead to a further improvement of image quality [11].

Solid-state digital PET detectors use a novel combination of lutetium-based scintillator crystal arrays with a silicon photomultiplier, which improves intrinsic sensitivity and temporal resolution [12]. These novel detector elements were made available clinically with the introduction of integrated digital PET/MR in 2013 [13]. Several technical and clinical studies showed a superior performance of digital compared to analog detector systems [14]. While PET/MR today is mainly limited to academic environments, silicon-based digital detector technology became available to PET/CT in the beginning of 2017, paving the way for a dissemination of this technique into the clinical field worldwide. A first study carried out in a mixed population of cancer patients showed an improved performance of digital PET/CT with regard to pathologic and physiologic structures [12].

The purpose of our study was to evaluate different reconstruction algorithms on the latest-generation digital PET/CT scanner and to identify the optimal reconstruction method for the quantitation of histopathologically confirmed lung cancer.

## Methods

### Patients

The inclusion criteria for this retrospective study were patients (a) with a histopathologically confirmed lung cancer regardless of tumor size who were (b) referred to our department for initial staging with clinically indicated 18F-FDG PET/CT between March and November 2017 (c) with written informed consent for the scientific use of medical data. This study was approved by the local ethics committee. The study was conducted in compliance with ICH-GCP-rules and the Declaration of Helsinki.

### 18F-FDG PET/CT imaging protocol

All patients underwent a PET/CT on a certified novel digital detector scanner (GE Discovery Molecular Insights - DMI PET/CT, GE Healthcare, Waukesha, WI). A body mass index (BMI)-adapted 18F-FDG dosage regimen was used, based on recommendations made by a previous study utilizing the same digital PET detector system [14]: A dose of 1.5 MBq/kg body weight was injected for patients with a BMI of < 20 kg/m^2^, 2 MBq/kg body weight for patients with a BMI of 20–24.5 kg/m^2^, and 3.1 MBq/kg body weight for patients with a BMI > 24.5 kg/m^2^, however, without exceeding a maximum injected 18F-FDG dose of 320 MBq. Participants fasted for at least 4 h prior to the scan, and blood glucose levels were below 160 mg/dl at the time of 18F-FDG injection. A CT scan was obtained from the vertex of the skull to the mid-thighs and used for attenuation correction purposes as well as for anatomic localization of 18F-FDG uptake. The CT scan was acquired using automated dose modulation (range 15–100 mA, 120 kV). Immediately after the CT, a PET scan was acquired covering the identical anatomical region. The FDG uptake time was set to 60 min. The PET acquisition time was 2.5 min per bed position, with 6–8 bed positions per patient (depending on patient size), with an overlap of 23% (17 slices). The PET was acquired in 3D mode and the slice thickness was 2.79 mm.

### PET reconstructions

After the PET acquisition, raw data were reconstructed with seven different reconstruction settings per patient; two reconstructions were using OSEM with two iterations, 24 subsets, and 6.4-mm Gaussian filter (1) with TOF (OSEM_TOF_; VUE Point FX, GE Healthcare) and (2) with TOF and point spread function modelling (OSEM_PSF_; Vue Point FX with SharpIR, GE Healthcare). Five reconstructions used BSREM (Q.Clear, GE Healthcare) with incremental *β-*values of (3) 350, (4) 450, (5) 600, (6) 800, and (7) 1200, respectively. All datasets were reconstructed with a 256 × 256 pixel matrix. The rationale for choosing the abovementioned reconstructions was twofold: first, to explore the broad range of reconstruction capabilities of the system and second, to cover different clinical scenarios: While OSEM_PSF_ represents the latest reconstruction technique used on many analog PET/CT systems, OSEM_TOF_ is used in clinical multicenter studies for the purpose of inter-scanner harmonization. BSREM on the other hand represents a full convergence algorithm, which has the potential to become a clinical standard in the future, at least for digital scanners [15, 16]. BSREM incorporates a penalty function which specifically suppresses noise fraught image solutions during the iteration process. As these are eliminated as options for the subsequent iterations, the number of iterations can be increased without detriment of increasing noise [17]. This penalization factor (i.e., *β*-value) represents the only user-input variable. The relative difference penalties for BSREM used in our study were chosen based upon preliminary testing.

### Subjective imaging analysis

A total of 315 reconstructed PET datasets (45 patient studies, each with 7 different reconstructions) were evaluated by two readers (M.M. and M.W.H., with 5 and 11 years of experience in chest radiology, respectively) blinded to the reconstruction method used. All scans were reviewed independently on a dedicated workstation (Advantage Workstation, Version 4.6; GE Healthcare) and in random order. Readers were blinded to any clinical information, except the presence of a primary lung tumor. In case of discrepancy of image rating, a final decision was made by consensus including a third reader.

The readers first rated the general image quality; for this purpose, datasets were viewed using maximum intensity projection (MIP) of PET and axial views with reformatted sections. The two readers evaluated the general image quality of each reconstructed image dataset using a 5-point Likert scale: 1, poor; 2, reasonable; 3, good; 4, very good; and 5, excellent quality. After that, the readers evaluated the images with regard to image sharpness and lesion conspicuity using another 5-point Likert scale, as suggested previously [18, 19]. For image sharpness, the readers rated as follows: 1, inadequate image with severe blurring; 2, diagnostically relevant image blurring; 3, diagnostically irrelevant image blurring; and 4, good images with minimal blurring; and 5, clear, excellent images. For lesion conspicuity, the readers rated as follows: 1, very poor conspicuity of lesion circumference; 2, poor conspicuity, < 25% of the lesion circumference clearly definable; 3, fair conspicuity, 25–50% of the lesion circumference definable; 4, good conspicuity, 50–75% of the lesion circumference definable; and 5, excellent conspicuity, > 75% of the lesion circumference definable, as previously described [14]. Finally, the readers were asked to choose the preferred reconstruction on a per-patient level, therefore reviewing all seven MIP PET images of a given patient side-by-side.

### Quantitative imaging analysis

Quantitative analyses were performed by a third reader (M.M.) in a separate reading session. The maximum standardized uptake value (SUV_max_) of each primary lung tumor was recorded using a standard volume of interest (VOI) tool. Herewith, the VOI was automatically propagated to cover exactly the same volume in all seven different reconstruction datasets. Moreover, background SUVs were assessed in the right lobe of the liver (parenchymal organ background) and within the descending aorta (bloodpool background) at the level of the carina, with 4.0-cm- and 1.0-cm-diameter spherical VOIs, respectively. Only liver parenchyma with normal appearance on both PET and CT was used as a reference. The mean standardized uptake value (SUV_mean_) and the standard deviation of the standardized uptake value (SUV_SD_) within the VOIs were recorded in both backgrounds for all reconstructions. Based on these measurements, a signal-to-background ratio (SBR) was calculated for each lung tumor, defined as the lung lesions’ SUV_max_ divided by the SUV_mean_ in the descending aorta. The liver SUV_SD_ was used as a measure of noise. Tumor signal-to-noise ratio (SNR) was defined as the lesions’ SUV_max_ divided by the liver SUV_SD_. Further, a contrast-to-background ratio (CBR) was calculated, defined as the (lung lesions’ SUV_mean_ − the SUV_mean_ in the descending aorta) divided by the SUV_mean_ in the descending aorta. And finally, contrast-to-noise ratio (CNR) was measured, defined as the (lung lesions’ SUV_mean_ − the SUV_mean_ in the descending aorta) divided by the liver SUV_SD_.

### Statistical analyses

Categorical variables are expressed as proportions, and continuous variables are presented as mean ± standard deviation or median (range), depending on the distribution of values. Qualitative image ratings (i.e., general image quality, image sharpness, and lesion conspicuity) were analyzed with the Friedman test separately, comprising all reconstruction algorithms and BSREM only. Further, qualitative image ratings (i.e., general image quality, image sharpness, lesion conspicuity, and preferred reconstruction per patient) were compared between patients with a low (i.e., ≤ 2.0 MBq/kg body weight; *n* = 25) and a high (i.e., > 2.0 MBq/kg body weight; *n* = 20) 18F-FDG dosage exam using Mann-Whitney *U* test. Since all quantitative SUV_max_ values were distributed normally, statistical differences were assessed using repeated measures analysis of variances (ANOVA) with post hoc Bonferroni corrections to adjust for multiple comparisons. Analyses were carried out using SPSS release 23.0 (IBM Corporation, Armonk, NY, USA) and MedCalc version 15.8 (MedCalc Software, Ostend, Belgium). A two-tailed *p* value of < 0.05 was considered to indicate statistical significance.

## Results

A total of 45 patients (16 female, 29 male, mean age 68 ± 10 years) referred for the initial staging of lung cancer with 18F-FDG PET/CT participated in our study. Patients had non-small cell lung cancer (NSCLC; *n* = 41), small cell lung cancer (SCLC; *n* = 3), and mixed NSCLC/SCLC (*n* = 1). Further demographic information including lung cancer stages according to the 8th Edition Lung Cancer Stage Classification [20] is given in Table 1.

**Table 1:** Demographic data of study subjects (*n* = 45)

Female/male, *n* (%)	16 (36%)/29 (64%)
Age, years	68 ± 10 (47–83)
Body weight, kg	71 ± 17 (39–114)
Body height, m	1.71 ± 0.1 (1.49–1.87)
BMI, kg/m^2^	24.3 ± 4.8 (15.0–36.8)
Blood glucose level at time of injection, mg/dl	101 ± 17 (67–157)
Injected tracer activity, MBq	175 ± 73 (85–318)
PET/CT scan post injection time, min	62 ± 9 (51–97)
Lung tumor localization, *n* (%)	
Peripheral	29 (64%)
Peri-hilar	16 (36%)
Lung cancer stage, *n* (%)^a^	
I	4 (10%)
II	5 (12%)
III	12 (29%)
IV	20 (49%)

Values are given as absolute numbers and percentages in parenthesis or mean ± standard deviation (range)

*BMI* body mass index, *MBq* mega-Becquerel, *PET* positron-emission tomography

^a^Stages for all patients with NSCLC (*n* = 41); according to the 8th Edition Lung Cancer Stage Classification [20]

### Subjective image quality

**Fig. 1:**
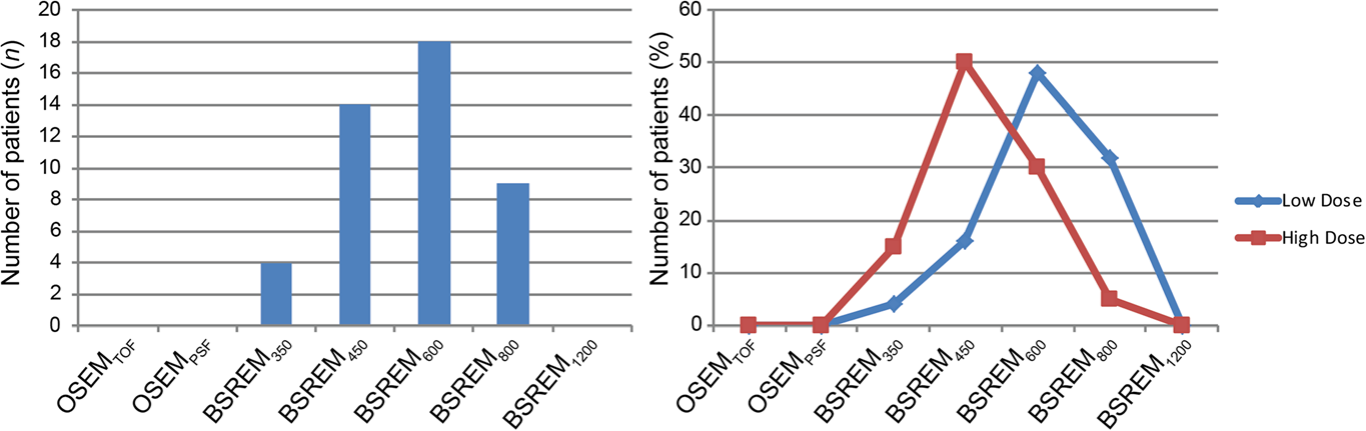
Absolute frequency distribution of preferred reconstruction algorithms for lung cancer assessment as rated by the readers, including the ratings for all study subjects (**a**): BSREM_600_ was chosen most frequently as the preferred reconstruction algorithm by the readers, followed by BSREM_450_, BSREM_800_, and BSREM_350_. When comparing the relative frequency distribution of preferred reconstruction algorithms (**b**) for high-18F-FDG-dosage regimen (> 2.0 MBq/kg body weight; *n* = 20 patients) and low-dosage regimen (≤ 2.0 MBq/kg body weight; *n* = 25 patients), a significant shift of the preferred image reconstruction algorithm from BSREM_450_ to BSREM_600_ was observed (*p* <  0.05)

The results of the subjective image assessment including all study subjects are given in Table 2. General image quality was rated significantly different among all different reconstruction algorithms as well as among BSREM using different *β*-values only (both *p* <  0.001). Similar differences were observed for image sharpness and lesion conspicuity (all *p* <  0.001). BSREM_600_ was assigned the highest score for general image quality, image sharpness, and lesion conspicuity. Accordingly, BSREM_600_ was chosen most frequently as the preferred reconstruction algorithm by the readers, i.e., in 18/45 (40%) cases, followed by BSREM_450_ in 14/45 (31%), BSREM_800_ in 9/45 (20%), and BSREM_350_ in 4/45 (9%) cases (Fig. 1).

**Table 2:** Results of subjective PET image quality ratings for different reconstruction algorithms. Italicized numbers are the reconstructed datasets yielding the highest score for each assessed parameter

Reconstruction	General image quality		Image sharpness		Lesion conspicuity	
	Mean	SD	Mean	SD	Mean	SD
OSEM_TOF_	3.0	0.6	2.1	0.6	2.1	0.6
OSEM_PSF_	3.9	0.5	3.3	0.7	3.2	0.6
BSREM_350_	3.8	0.9	4.3	0.6	4.1	0.8
BSREM_450_	4.4	0.6	4.5	0.5	4.4	0.6
BSREM_600_	*4.8*	0.4	*4.6*	0.5	*4.5*	0.5
BSREM_800_	4.8	0.4	4.2	0.5	4.3	0.7
BSREM_1200_	4.1	0.5	3.3	0.6	3.6	0.6

*BSREM* block sequential regularized expectation maximization, *OSEM* ordered subset expectation maximization, *PSF* point spread function modelling, *TOF* time of flight

### Effect of administered 18F-FDG dose on image quality

Table 3 demonstrates image quality ratings according to the administered dose of 18F-FDG. Significant differences between patients with high-dose regimen (i.e., > 2.0 MBq/kg body weight; *n* = 20) and low-dose regimen (i.e., ≤ 2.0 MBq/kg body weight; *n* = 25) are indicated (see Table 3). A statistically significant shift of the preferred reconstruction algorithm towards higher *β*-values was observed in patients with low-dose regimen compared to patients with high-dose regimen (*p* <  0.05, Fig. 1). In patients with high-dose regimen, BSREM_450_ was chosen most frequently as the preferred reconstruction algorithm by the readers, i.e., in 10/20 (50%) cases, followed by BSREM_600_ in 6/20 (30%), BSREM_350_ in 3/20 (15%), and BSREM_800_ in 1/20 (5%) cases. On the other hand, in patients with low-dose regimen, BSREM_600_ was chosen most frequently as the preferred reconstruction algorithm by the readers, i.e., in 12/25 (48%) cases, followed by BSREM_800_ in 8/25 (32%), BSREM_450_ in 4/25 (16%), and BSREM_350_ in 1/25 (4%) cases.

**Table 3:** Results of subjective PET image quality ratings for different reconstruction algorithms in a subanalysis for patients with high-dose (≥ 2.0 MBq/kg (*n* = 20 patients of study group)) and low-dose (≤ 2.0 MBq/kg (*n* = 25 patients of study group)) injection regimen of 18F-FDG. Italicized numbers are the reconstructed datasets yielding the highest score for each assessed parameter

Reconstruction	General image quality		Image sharpness		Lesion conspicuity	
	High dose	Low dose	High dose	Low dose	High dose	Low dose
OSEM_TOF_	3.1	2.9	2.2	2.0	2.3	1.9*
OSEM_PSF_	3.8	3.9	3.5	3.0*	3.3	3.0
BSREM_350_	4.0	3.5*	4.5	4.0**	4.2	4.0
BSREM_450_	4.6	4.1**	*4.7*	4.3**	4.6	4.2*
BSREM_600_	*4.8*	4.7	4.7	*4.6*	*4.6*	*4.5*
BSREM_800_	4.8	*4.8*	4.1	4.4	4.3	4.0
BSREM_1200_	4.2	4.0	4.2	3.4	3.7	3.6

Data are presented as mean

*BSREM* block sequential regularized expectation maximization, *OSEM* ordered subset expectation maximization, *PSF* point spread function modelling, *TOF* time of flight

* *p* value < 0.05, ** *p* value < 0.01

### Quantitative image assessment

The results of the quantitative analysis including SUV_max_, SBR, SNR, CBR, and CNR in the differently reconstructed datasets are given in Table 4. SUV_max_ and SBR were highest in BSREM_350_ and decreased with incremental *β-*values, whereas there was a continuous increase of SNR with increasing *β-*values. In Table 5**,** the median relative differences of SUV_max_ comparing all reconstruction algorithms are given, including *p* values for pairwise comparison.

**Table 4:** Results of quantitative PET image assessment for different reconstruction algorithms including maximum standardized uptake value (SUV_max_) of the primary lung tumor, tumor signal-to-background ratio (SBR), tumor signal-to-noise ratio (SNR), contrast-to-background ratio (CBR), and contrast-to-noise ratio (CNR). Italicized numbers are the reconstructed datasets yielding the highest values for given parameters

	OSEM		BSREM				
	OSEM_TOF_	TOF_PSF_	BSREM_350_	BSREM_450_	BSREM_600_	BSREM_800_	BSREM_1200_
SUV_max_							
Mean	11.9	12.7	*15.0*	14.5	14.0	13.4	12.8
Median	11.7	12.8	*14.3*	14.2	13.8	13.4	12.9
Range	3.6–25.2	3.5–26.7	*4.0–30.4*	3.8–29.9	3.6–29.2	3.4–28.4	3.2–27.5
SBR							
Mean	6.9	7.4	*8.8*	8.5	8.1	7.6	7.3
Median	6.7	7.3	*8.7*	8.4	7.9	7.7	7.3
Range	1.9–12.8	1.8–13.6	*2.1–16.6*	1.9–16.0	1.8–15.3	1.7–14.6	1.6–13.8
SNR							
Mean	41.7	48.3	51.1	55.4	62.6	70.2	*82.3*
Median	38.9	43.5	48.5	51.7	58.0	64.3	*75.6*
Range	11.6–81.1	11.9–92.1	11.1–101.4	11.9–103.1	13.3–116.7	14.9–135.3	*16.7–161.9*
CBR							
Mean	3.1	3.4	*4.1*	4.0	3.8	3.6	3.4
Median	3.1	3.4	*3.8*	3.8	3.5	3.4	3.3
Range	0.2–6.5	0.1–7.0	*0.2–8.2*	0.1–7.8	0.1–7.6	0.1–7.2	0.04–6.8
CNR							
Mean	18.8	22.2	23.8	26.0	29.3	32.8	*38.3*
Median	18.1	20.3	21.6	23.8	26.7	29.4	*34.0*
Range	1.0–40.0	0.9–44.9	1.0–49.3	0.9–50.0	0.8–56.7	0.6–65.7	*0.4–78.4*

*BSREM* block sequential regularized expectation maximization, *OSEM* ordered subset expectation maximization, *PSF* point spread function modelling, *TOF* time of flight

**Table 5:** Median differences (range) of tumor maximum standardized uptake value (SUV_max_) using different reconstruction algorithms are displayed in the upper right half of the table. The lower left half of the table shows *p* values of pairwise comparisons of different reconstructions

	Difference^a^ of SUV_max_						
	OSEM_TOF_	TOF_PSF_	BSREM_350_	BSREM_450_	BSREM_600_	BSREM_800_	BSREM_1200_
OSEM_TOF_	–	+ 6.8% (− 4.2 to + 22.0%)	+ 28.0 (+ 6.9 to + 73.5%)	+ 23.4% (+ 4.1 to + 66.6%)	+ 18.5% (− 0.3 to + 61.2%)	+ 13.4% (− 5.0 to + 54.0%)	+ 8.2% (− 11.9 to + 41.3%)
OSEM_PSF_	< 0.001	–	+ 17.7% (− 2.7 to + 42.3%)	+ 14.1% (− 5.3 to + 36.6%)	+ 9.3% (− 8.6 to + 32.2%)	+ 6.4% (− 12.4 to + 26.3%)	+ 0.8% (− 18.0 to + 15.8%)
BSREM_350_	< 0.001	< 0.001	–	− 3.6% (− 12.3 to + 6.7%)	− 6.8% (− 24.0 to − 1.3%)	− 10.2% (− 34.0 to − 3.9%)	− 15.1% (− 31.4 to − 5.9%)
BSREM_450_	< 0.001	< 0.001	< 0.001	–	− 3.5% (− 13.2 to − 1.0%)	− 7.5% (− 31.0 to − 2.7%)	− 12.5% (− 21.9 to − 4.8%)
BSREM_600_	< 0.001	< 0.001	< 0.001	< 0.001	–	− 3.9% (− 26.7 to − 1.4%)	− 9.0% (− 17.5 to − 3.6%)
BSREM_800_	< 0.001	< 0.001	< 0.001	< 0.001	< 0.001	–	− 4.8% (− 10.6 to + 16.7%)
BSREM_1200_	< 0.001	NS	< 0.001	< 0.001	< 0.001	< 0.001	–

Post hoc pairwise comparison with Bonferroni-adjustment for multiple comparison

*BSREM* block sequential regularized expectation maximization, *OSEM* ordered subset expectation maximization, *PSF* point spread function modelling, *TOF* time of flight, *NS* not significant

^a^Calculated as (SUV_max_ in each dataset [i.e., 2nd row] − SUV_max_ reference [i.e., 1st column]) × 100/(SUV_max_ reference)

**Fig. 2:**
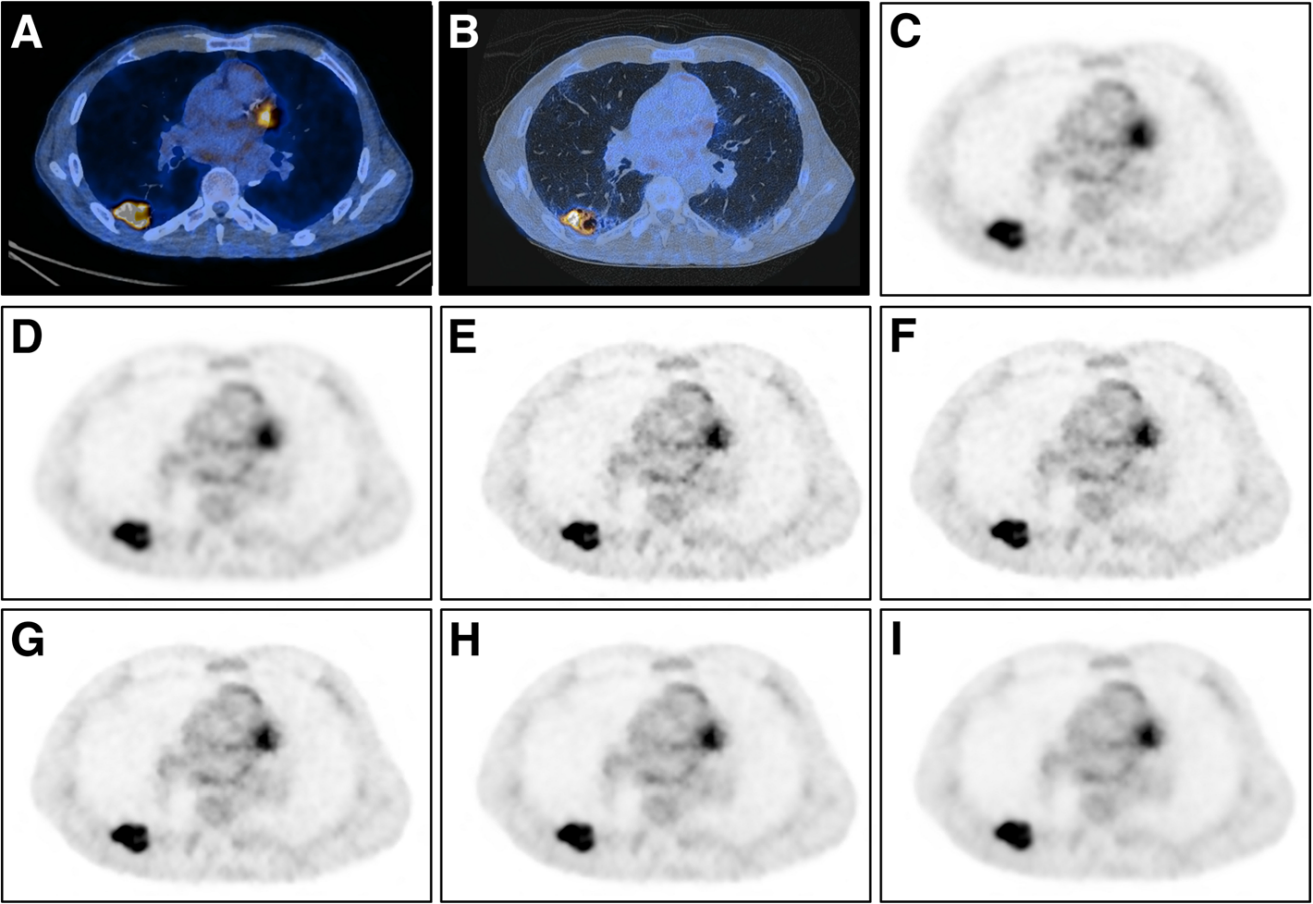
Representative images of a 66-year-old man with a body mass index of 20.7 kg/m^2^ and 59 kg body weight who underwent 18F-FDG PET/CT for staging of lung cancer. The patient was injected with 117.9 MBq of 18F-FDG (i.e., 2.0 MBq/kg body weight), according to the BMI-adapted dosage protocol developed for digital PET [14]. Co-registered PET/CT images (**a**, **b**) show a highly 18F-FDG-avid tumor in the right lower lobe, which was confirmed as adenocarcinoma after wedge resection. Axial PET images are given in **c–i**, showing OSEM_TOF_ (**c**), OSEM_PSF_ (**d**), BSREM_350_ (**e**), BSREM_450_ (**f**), BSREM_600_ (**g**), BSREM_800_ (**h**), and BSREM_1200_ (**i**)

**Fig. 3:**
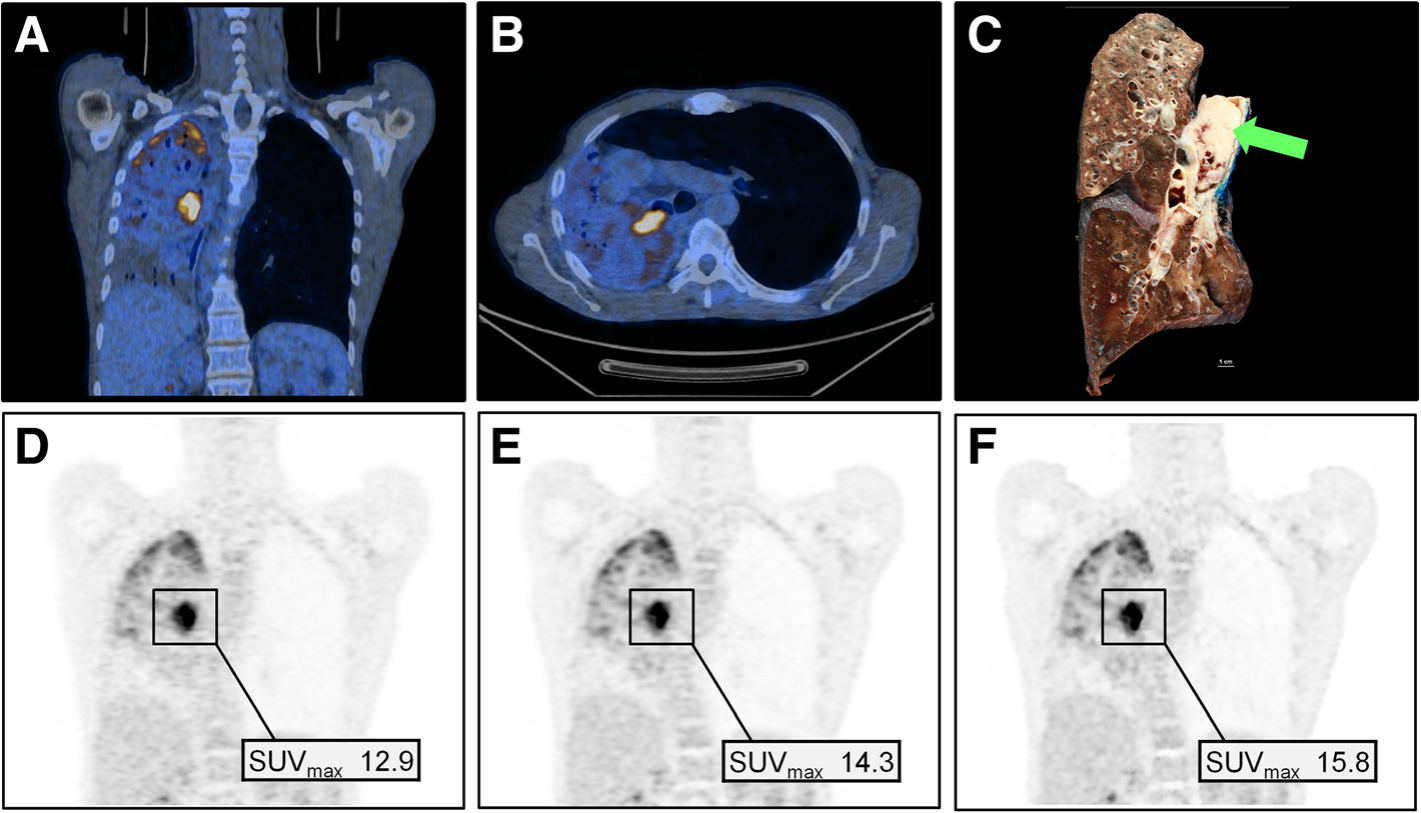
Representative images of a 53-year-old man with a body mass index of 19.1 kg/m^2^ and 66 kg body weight who underwent 18F-FDG PET/CT for staging of lung cancer. The patient was injected with 99.8 MBq of 18F-FDG (i.e., 1.5 MBq/kg body weight). Coronal and axial co-registered PET/CT images (**a**, **b**) show a highly 18F-FDG-avid tumor invading the right main bronchus, which was confirmed as squamous cell carcinoma (arrow) after right-sided pneumonectomy (**c**). Coronal PET images show OSEM_TOF_ (**d**), OSEM_PSF_ (**e**), and BSREM_600_ (**f**) reconstruction together with SUV_max_ of the primary tumor

Representative images of study subjects undergoing 18F-FDG PET/CT for staging of lung cancer are given in Figs. 2 and 3.

## Discussion

This study sought to evaluate the impact of different PET reconstruction algorithms on image quality and quantitative parameters in patients with histopathologically confirmed lung cancer using a latest-generation silicon-based digital detector PET/CT scanner. The major findings of our study are as follows: (1) BSREM reconstruction algorithms lead to an increased image quality, image sharpness, and tumor lesion conspicuity compared to OSEM; (2) adjusting *β*-values to the injected 18F-FDG activity allows for an individual dose-based optimization of image quality of PET images; and (3) BSREM reconstruction leads to a significant increase of SUV_max_, which is most prominent with lower *β*-values (e.g., 350).

PET/CT using 18F-FDG as radiotracer has evolved to be the most important cross-sectional imaging modality for whole-body staging of patients with lung cancer in recent years and is recommended by various international guidelines [6, 21]. There is, however, an inherent relatively low spatial resolution [7] as well as a generally low signal-to-noise ratio of PET [8]. This is why improving the image quality of PET is an ongoing subject of research and, besides new hardware features such as TOF acquisition [9], advanced PET data reconstruction methods are being developed. For example, iterative reconstruction methods such as OSEM have been widely adopted instead of the initially used filtered back projection, leading to an overall image improvement [10, 22]. Based on raw data sinograms, OSEM repeatedly iterates different possibilities in order to find the most probable image. Thereby, with each iteration step, an image with a greater likelihood of describing the measured data is achieved. The main disadvantage of OSEM, however, is the impossibility to run iterations to full convergence, because the image noise increases with each iteration, leading to rather unacceptable image quality before full convergence is reached [23, 24]. Therefore, OSEM is stopped after a predefined number of iterations, resulting in under-converged images. As a main consequence, this leads to an underestimation of the true SUV.

BSREM on the other hand, as a fast and globally convergent reconstruction algorithm, may increase the accuracy of lesion quantitation compared to OSEM, with a particular improvement in cold background regions such as the lungs as indicated in previous studies using NEMA and anthropomorphic phantom data [16]. Moreover, in a clinical setting, Teoh et al. showed that BSREM may significantly increase the SUV_max_ and increase signal-to-background/noise of lung lesions [25]. These observations are in line with the results of our study, e.g., a median increase of lung tumor SUV_max_ by 9.3% or 17.7% with BSREM_600_ or BSREM_350_, respectively, compared with OSEM_PSF_, or by 18.5% or 28.0% with BSREM_600_ or BSREM_350_, respectively, compared with OSEM_TOF_.

It is understood that increased quantitative accuracy in PET does not necessarily translate into an improvement of clinical readings. We therefore included in our study performance assessments of readers to complement the quantitative approach and enable a meaningful clinical implication. We could show that several aspects of reading lung cancer PET exams are enhanced with BSREM, such as lesion conspicuity and image sharpness. Indeed, in all 45 patients, a BSREM reconstruction was selected as preferred reconstruction for image assessment by the readers. An “intermediate” *β*-value (i.e., 450–600) seems to be ideal for lung cancer assessment and was selected in most cases. This is paralleled by the observation that by applying incrementally higher *β*-values, a steady increase of signal-to-noise ratio comes at the expense of reduced tumor signal-to-background ratio as a quantitative term but also at the expense of image sharpness as qualitative/subjective term.

As expected based on the objectives of BSREM, we observed a significant shift of the selected “preferred image reconstruction” towards higher *β*-values (i.e., from 450 to 600) in patients who received lower 18F-FDG doses (< 2 MBq/kg) compared with patients who received higher doses (> 2 MBq/kg). This observation reflects the apparent ability of BSREM to balance image quality and noise levels according to the injected dose and/or patient BMI by choosing different *β*-values, with higher *β*-values appearing more appropriate for patients with lower 18F-FDG doses. Hence, the appropriate selection of this relative difference penalty may become a valuable tool for adjusting PET image quality on a per-patient base, allowing both for a more patient-tailored PET imaging and for maintaining adequate image quality while reducing the dose. Notably, it is yet not known at which threshold particularly small lesions are lost with increasing *β*-values using BSREM reconstruction [25].

While limiting the 18F-FDG dose may not seem to be overly important in patients with lung cancer, dose reduction in PET in general is a worthwhile goal to achieve. This is particularly true in patients with diseases requiring repeat examinations such as lymphoma and especially for young patients who have a comparably high life expectancy. In this patient group, the imaging modality with the lowest achievable absorbed radiation dose per imaging study is desired. Future studies on, e.g., lymphoma patients may further refine protocols to let BMI-based dose adaption become reality also for this patient group, who are at particular stochastic risk for potential adverse radiation effects.

We acknowledge that our study has some limitations. First, a clinical reader assessment as the one we performed might carry an inherent bias since it is virtually impossible to totally blind readers to the image “appearance” of different reconstruction algorithms. Second, analyses of tumor SUV were restricted to measurement of SUV_max_, which is the single most important PET parameter in clinical care. Further evaluation of corrected SUV would possibly alter the results. Third, we did not stratify our analyses by tumor size. Fourth, we used only a small range of possible *β*-values based on pretests. Fifth, the FDG dose regimen was based on BMI and body weight and—while having been specifically developed for digital detector PET—may differ from other protocols used on analog detector scanners in conjunction with BSREM. Sixth, the number of patients in this single-center study is comparably small, and therefore, conclusions drawn from the present analysis await further proof in larger (and ideally multi-centric) observations. Future studies are also warranted to assess the impact of BSREM on diagnosis, clinical management, and patient outcome.

## Conclusions

In conclusion, BSREM reconstruction algorithm used in digital detector PET leads to significant increases of lung tumor SUV_max_, signal-to-background ratio, and signal-to-noise ratio, which translates into a higher image quality, tumor conspicuity, and image sharpness.

## Abbreviations

18F-FDG: 18F-Fluorodeoxyglucose
BMI: Body mass index
BSREM: Block sequential regularized expectation maximization
CT: Computed tomography
MIP: Maximum intensity projection
NSCLC: Non-small cell lung cancer
OSEM: Ordered subset expectation maximization
PET: Positron-emission tomography
SCLC: Small cell lung cancer
SUV_max_: Maximum standardized uptake value
SUV_mean_: Mean standardized uptake value
SUV_SD_: Standard deviation of standardized uptake value
TOF: Time of flight
VOI: Volume of interest
